# Analysis of Amino Acid Changes in the Fusion Protein of Virulent Newcastle Disease Virus from Vaccinated Poultry in Nigerian Isolates

**DOI:** 10.1155/2022/9979683

**Published:** 2022-10-31

**Authors:** Olubukola O. Funsho-Sanni, Elijah E. Ella, Lawal D. Rogo, Olufunsho S. Sanni, Helen I. Inabo, Sodangi A. Luka, Ismaila Shittu

**Affiliations:** ^1^Department of Microbiology, Faculty of Life Sciences, Ahmadu Bello University, P.M.B.06, Zaria, Kaduna, Nigeria; ^2^Department of Medical Laboratory Science, Faculty of Allied Health Sciences, College of Health Sciences Bayero University Kano, P.M.B.3011, Kano, Nigeria; ^3^Center for Integrated Health Programs, Kikuyi Close, Wuse 904101, Abuja, Nigeria; ^4^Department of Zoology, Faculty of Life Sciences, Ahmadu Bello University, P.M.B.06, Zaria, Kaduna, Nigeria; ^5^National Veterinary Research Institute, P.M.B.01, Vom, Plateau, Nigeria

## Abstract

The roles of fusion gene in the virulence of Newcastle disease virus are well established, but the extent of its variation among the XIV, XVII, and XVIII genotypes reported in Central Africa and West Africa has until recently been understudied. In this study, virulent Newcastle disease virus (vNDV) was isolated from dead chickens among vaccinated flocks between March and April 2020. Fusion (F) gene was sequenced and analysed for characterization and information about genetic changes. Many substitutions were observed along the region and some of their functions are yet to be determined. Results showed that all study isolates have virulent cleavage site sequence 112-RRRKR-116/F117 and clustered within genotype XIVb. Sequence analysis showed K78R mutation in the A2 antigenic epitope in all isolates and more along the F-gene which varied in some instances within the isolates. Mutation in this A2 antigenic epitope has been reported to induce escape mutation to monoclonal antibodies generated using the NDV LaSota strain. The range of percentage nucleotide and amino acid homology between the study isolates and commercially available vaccine strains is 81.14%–84.39% and 0.175–0.211, respectively. This report provides evidence of vNDV among vaccinated chicken flock and molecular information about circulating vNDV strains in Kano State, Nigeria, which is useful for the development of virus matched vaccines. Newcastle disease (ND) surveillance and molecular analysis of circulating strains in this region should be encouraged and reported. Furthermore, ND outbreaks or cases among vaccinated poultry presented to veterinary clinics should be reported to the state epidemiologist. Nucleotide sequences were assigned accession numbers OK491971–OK491977.

## 1. Introduction

Newcastle disease (ND) is a globally reported viral disease affecting over 200 species of birds [1] primarily controlled by vaccination [2]. It is an Office International des Épizooties (OIE) notifiable disease [1]; however, only few countries report its incidence to OIE, especially in developing countries where the disease is enzootic [3] among vaccinated and unvaccinated poultry. ND has received extensive attention because of its ability to spread, high mortality, vaccine failure, and other economic losses associated. The aetiology of ND is virulent strain of Avian paramyxovirus type-1 (APMV-1) also known as Newcastle disease virus (NDV) of the genus *Orthoavulavirus* belonging to the family *Paramyxoviridae* and order *Mononegavirales* [1]. It is a negative sense, non-segmented, and single-stranded enveloped RNA virus [4].

NDV fusion (F) glycoprotein mediates fusion between the viral and host cellular membranes [5, 6]. It is synthesized as an unreactive F0 precursor, containing 1662 nucleotides (nt) coding for 553 amino acids (aa) with an approximate 55 kDa weight [7, 8]. F0 is proteolytically cleaved by specific host cellular proteases at the peptide bond between residues 116 and 117 forming two disulphide linked polypeptides, F1 and F2 which are 48–54 kDa and 10–16 kDa, respectively [9, 10]. There are several domains that have been identified throughout the length of these polypeptides important for viral fusion activity [11].

Although Newcastle disease (ND) is said to be enzootic in Nigeria, little information exists on the molecular epidemiology and the lineage distribution of the Newcastle disease virus (NDV) in the country [3] and there is paucity on reports of virulent Newcastle disease virus (vNDV) strains obtained from dead/sick vaccinated animals in the outpatient veterinary clinic. The importance of detection and pathotyping of NDV in understanding the epizootiology of the virus in any region cannot be overemphasized [12] especially with the growing need for evaluation of the efficacy of existing ND vaccines [13]. Presently, two live attenuated monovalent vaccines—LaSota and Komarov—are commercially available for the control of ND in intensively reared poultry in Nigeria, but there are reports of ND outbreak among vaccinated flocks. Knowledge of the increasingly evolving genetic variation of vNDV is important for developing genetically matched vaccines which can prevent ND vaccine failure and maybe future outbreaks in the country.

The aim of this study was to isolate and characterize Newcastle disease virus (NDV) full fusion (F) gene detected from dead chicken of vaccinated flock presented to a veterinary clinic for post-mortem examination in Kano State, Nigeria, during March and April, 2020. Details of amino acid mutation were noted, documented, and compared with previously reported vNDV isolates from Nigeria, West Africa, and commercially available NDV vaccine strains.

## 2. Materials and Methods

### 2.1. Animal Ethics Declaration

This study did not include the use of live chickens. International and national guidelines for the care and use of animals were followed by experts at the veterinary clinic during post-mortem examinations and sample collection.

### 2.2. Sample Collection

Pooled organ samples (proventriculus, spleen, and small intestine) and swabs (cloacal and tracheal) were collected in viral transport medium (VTM) from one hundred chicken cadavers with history of vaccination against NDV that was reported to a veterinary clinic located in Kano State metropolis during March-April 2020 from flocks presenting with respiratory discomfort, weakness, greenish diarrhoea, anorexia, high mortality and morbidity, and drop in egg production in layers characteristic of ND. The samples were labelled and transported immediately on ice and stored at −4°C until analysis was conducted.

Post-mortem examination was conducted on all specimens, and characteristic lesions were noted.

### 2.3. NDV Total Viral RNA Extraction, Reverse Transcription Polymerase Chain Reaction, and F-Gene Sequencing

All experiments were carried out according to standard protocol. Viral RNA was extracted directly from pooled organ samples and swabs using the Quick-RNA^TM^ Viral Kit (Zymo Research, USA) as specified in the product manual. Fifty samples were processed successfully.

NDV M-gene was detected using protocols described [14]. Two overlapping fragments covering 1662 bp of the full F-gene were amplified using two pairs of primers (Table 1). One-step RT-PCR was performed using One *Taq* one-step RT-PCR Kit (New England BioLabs ^Inc^). cDNA synthesis was achieved at 50°C for 30 minutes followed by incubation at 94°C for 15 minutes. RT-PCR was performed with 40 cycles of denaturing at 94°C for 30 seconds, annealing at 55°C for 1 minute, and extension at 68°C for 2 minutes with final extension at 68°C for 10 minutes, and PCR products were maintained at 4°C. Electrophoresis of PCR products was done on 1% ethidium bromide stained with 1.5% agarose gel at 90 volts and 120 Amps for 35 minutes and compared with a 100 bp DNA ladder, and amplified products were visualized under ultraviolet (UV) illumination using gel documentation system-image capture (Biometra, Germany).

The amplicons were sequenced by Macrogen Ltd (Netherlands), and sequences obtained were submitted on the NCBI database and assigned accession numbers OK491971–OK491977.

### 2.4. Phylogenetic Tree Construction and Evolutionary Distance Analysis

Nucleotide sequence alignment, editing, and analysis were done using the Bioedit software (7.2.5). Nucleotide sequence similarity and molecular phylogenetic analysis was computed on MEGA (11.0.). Inferred evolutionary F-gene sequence of the study isolates, some reported NDV isolates, and known vaccine strains was conducted by the maximum likelihood method based on JJT matrix-based method using 1000 bootstrap replicates.

## 3. Results

### 3.1. Characteristic Pathological Lesions on Organs Seen during Post-Mortem Examination

### 3.2. Molecular Detection of NDV

The overall M-gene detection rate by RT-PCR was 54% (27/50). A 121-bp fragment was amplified, and products of electrophoresis were visualized by ultraviolet (UV) trans-illumination.

**Figure 1:**
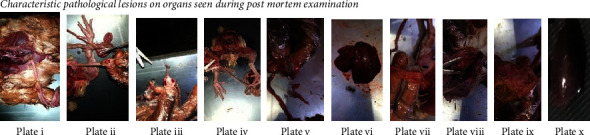
Plate i: cross-section of dissection of a layer; Plate ii-iii: haemorrhagic caeca tonsils (Peyer's patches); Plate iv: ecchymotic haemorrhage on the proventriculus; Plate v: haemorrhagic enteritis; Plate vi: congested and enlarged liver; Plate vii: haemorrhagic intestinal tract: Plate viii: haemorrhages on the trachea; Plate ix: inflamed and haemorrhagic proventriculus; Plate x: inflamed spleen.

F-gene amplification was successfully carried out in seven samples, and products of electrophoresis were visualized by UV trans-illumination (Figure 1). 1662 bp nucleotide sequences obtained were deposited in the GenBank (details are given in Table 2).

### 3.3. Phylogenetic Tree and Evolutionary Distance Analysis

**Figure 2:**
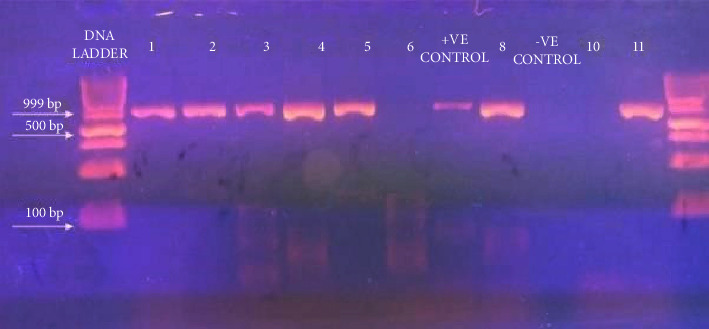
DNA Ladder, band showing 999 bp:-Lane 1-5, 8, and 11 positive samples, Lane 6 and 10 negative samples, lane 7 positive control, and lane 9 negative control.

Phylogenetic tree was constructed (Figure 2) using MEGA (11.0). The genetic relatedness of the study isolates, reference KU665482.1 LaSota.71.IR/2016, eight commercially available vaccine strains and NDV F-gene sequences obtained from GenBank database was inferred by phylogenetic analysis. All study isolates clustered around the newly classified genotype XIV (sub-genotype XIVb) in class II which is widely reported in Nigeria.

Nucleotide blast analysis shows a 99% nucleotide identity to virulent NDV MT543153 isolated in 2019 from backyard poultry in Niger (a country to the north border of Nigeria). The evolutionary divergence as nucleotide (nt) homology and amino acid (aa) homology between study isolates and commercially available vaccines is shown Tables 3 and 4.

### 3.4. Molecular Characterization and Mutational Analysis of the Functional and Antigenic Domains

The complete translated 553 fusion protein amino acid sequences obtained from the study isolates were used to compare their functional and antigenic domains relative to nine vaccine strains using KU665482.1 LaSota.71.IR/2016 as reference, six vNDV strains previously reported from Nigeria, and two vNDV strains isolated from West Africa(Tables 5–10). Notable substitutions around these regions were observed. Interestingly, among the research isolates, these substitutions were sometimes observed differently. Numbering system of amino acid (aa) was used to name the detected aa substitutions with respect to observed genetic variations.

**Figure 3:**
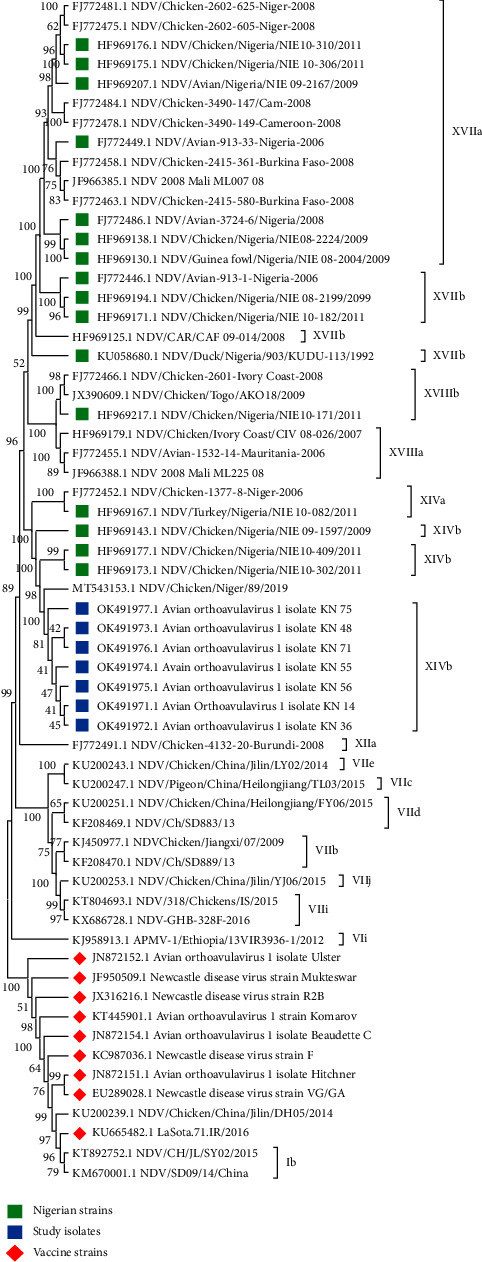
Molecular phylogenetic analysis based on the nucleotide sequences of the F-gene of study isolates, some reported NDV isolates and known vaccine strains using bootstrap consensus of 1000 replicates. The evolutionary history was inferred by using the Maximum Likelihood method and JTT matrix-based model [15]. The tree with the highest log likelihood (–20685.06) is shown. The percentage of trees in which the associated taxa clustered together is shown next to the branches. Initial tree(s) for the heuristic search were obtained automatically by applying Neighbor-Join and BioNJ algorithms to a matrix of pairwise distances estimated using the JTT model, and then selecting the topology with superior log likelihood value. This analysis involved 61 amino acid sequences. There were a total of 1662 positions in the final dataset. Evolutionary analyses were conducted in MEGA11 [16].

Along the hypervariable region (residues 1–31), 14 substitutions leading to P4K, P4E, A11V, A11E, L15Q, L28P, and A29T mutations were observed (Figure 3(a), Tables 5 and 7). In comparison to the LaSota reference strain KU665482, all isolates displayed a S31P of the signal peptide. Eight transmembrane domains have been reported[18] at residues 14–27, 15–25, 118–131, 120–128, 266–269, 429–432, 499–525, and 501–523. Compared to the LaSota strain, this region is highly non-conserved in all isolates except for 266–269 with no amino acid substitution (Figure 3(a)). The major epitopes involved in virus neutralization are conserved in all residues except for one amino acid substitution Lys AAG to Arg AGA (K78R) of the A2 neutralizing epitope identified in all isolates (Figure 3(a), Tables 5 and 7). However, nucleotide (nt) substitution occurred even in the conserved epitopes (Supplementary material (Available (here))) which shows disposition of these sites to aa mutation.

All seven isolates share the characteristic virulent motif ^112^R-R-R-K-R/F^117^ at the F0 cleavage site indicating that they are velogenic NDV strains (Figure 3(a)). G112R, Q114R, and G115K have been observed in study isolates (Tables 5, 7, and 8). Along the fusion peptide region (117–142), five aa substitutions, L117F, I118V, I121V, G124S, and I135V, are seen (Tables 5, 8, and 9). L117F and I118V are expected in the virulent furin-like molecule. In addition, the F protein has six highly conserved potential N-linked glycosylation sites Ng_1_–Ng_6_ [18] with sequence Asn (Asparagine)-X-Ser(Serine)/Thr(Threonine) (N-X-S/T) where X is any aa except proline and aspartate [19,20]. Amino acids at these sites were used and conserved in all NDV isolates of this research at residues 85NRT, 191NNT, 366NTS, 447NIS, 471NNS, and 541NNT. Hence, there was no loss of glycosylation site though there was one substitution compared to the LaSota strain at residue 191NKT (Figure 3(a)). However, nt substitutions occurred in different patterns at these regions among study isolates which resulted in same sense mutation.

Cysteine residues are important in the connection between F1 and F2 sub-units to maintain the F protein structure. Cysteine residues are conserved at positions 25, 27, 76, 199, 338, 347, 362, 370, 394, 399, 401, 424, 514, and 523 of the F protein in most NDV isolates [20]. Amino acids are used and conserved in all the isolates except for a unique point cysteine (C) to Serine substitution at residue 394 in OK491977 leading to loss of one cysteine residue. Several nt substitutions were observed in the region which resulted in same sense mutation (Figure 3(b), Tables 6 and 9).

The three heptad repeat regions HRa (143–185), HRb (268–299), and HRc (471–500) in the isolates [19] displayed 1, 5, and 6 aa substitutions, respectively, compared to the KU665482.1 LaSota.71.IR/2016 reference strain. These are K145N at HRa; N272Y, S278P, I285K, T288N, and N297K at HRb and N476T, N479D, E482A, R486N, K494R, and T498S at HRc. Notably, substitution I285K was seen only in OK491973 and OK491975. N297K was seen in isolate OK491973 only. N476T was observed only in isolate OK491976. Interestingly, T498S was observed in all study isolates and MT543153 NDV/chicken/Niger/89/2019 but not in any other isolate included in the phylogenetic tree analysis even those isolated previously from Nigeria or Africa (Figure 3(b), Tables 6, 9, and 10).

The effective B-cell epitope regions 157–171 involved in virus neutralization are surface-exposed amino acids which may cause antigenic difference between the vaccine and wild strains [19]. Amino acid sequence analysis of the isolates shows no aa substitutions within this region (Figure 3(a))

## 4. Discussion

NDV management in Africa is complicated [21]. The economic impact of ND among vaccinated and unvaccinated commercial and backyard poultry in Nigeria is significant. This translates even to point of sale of live birds (the sale of poultry product in Nigeria is unregulated and open) where traders record death of chickens and other birds due to disease in live bird markets. Research focus has largely been on NDV isolated from wild birds and their role in the epidemiology of the disease and molecular analysis using the full F-gene for pathotyping and lineage distribution studies. However, despite mass administration of vaccines, there are reports of vaccine failure and high mortality among vaccinated flocks [22], sub-optimal protection levels among vaccinated flocks [23], viral evolution and identification of new genotypes [19, 21], and significant antigenic distance between circulating and vaccine strains [24]. Despite huge patronization of veterinary clinics (for drugs and vaccines) and consultancy by poultry producers, to the best of our knowledge, this is the first report of isolation of vNDV from dead chickens from vaccinated flock presented to a veterinary clinic in Nigeria for post-mortem examination. This research also provides details of F-gene sequence of seven clinical vNDV isolated from Kano State, Nigeria.

**Figure 4:**
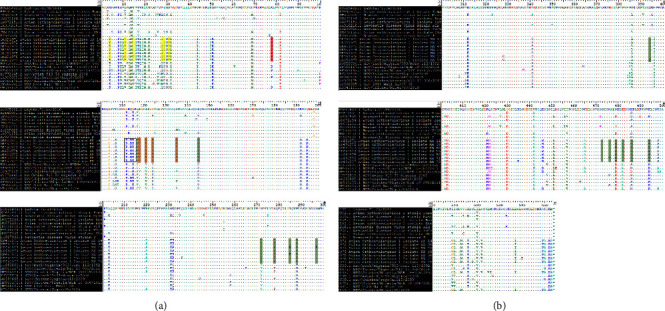
(a) Mutation profile along fusion gene of Newcastle disease virus (NDV). Figures show mutational substitution of study isolates in comparison with vaccine strains LaSota KU665482, Komarov KT445901, Muketswar JT950509, Beaudette C JN872154, Hitchner JN872151, R2B JX316216, VG/GA EU289028, Ulster JN872152, F KC987036; Six Nigerian isolates KU058680, HF969167, HF969143, FJ772449, HF969143, HF969175; Togo isolate JX390609; Mali isolate JF966385. Yellow colour-mutations along the Hypervariable region/Signal peptide; red colour-mutations along the A2 antigenic epitope; grey border line-cleavage site region; orange colour-mutation along fusion peptide region; green colour- mutation along the hypervariable region. (b) Mutation profile along fusion gene of Newcastle disease virus (NDV). Figures show mutational substitution of the seven Nigerian study isolates in comparison with vaccine strains LaSota KU665482, Komarov KT445901, Muketswar JT950509, Beaudette C JN872154, Hitchner JN872151, R2B JX316216, VG/GA EU289028, Ulster JN872152, F KC987036; Six Nigerian isolates KU058680, HF969167, HF969143, FJ772449, HF969143, HF969175; Togo isolate JX390609; Mali isolate JF966385. Green colour-mutation along the hypervariable region.

Seven genotypes (I, II, IV, VI, XIV, XVII, and XVIII) have been reported to circulate in Nigeria suggesting high genetic diversity of NDV for one country [25]. A phylogenetic tree was constructed based on the full F gene of the research isolates to determine their genotype. Isolates clustered among genotype XIVb in class II similar to earlier reports of newly classified genotypes XIV, XVII, and XVIII are identified as the circulating strains in Central Africa and West Africa [2, 21, 24]. The NDV isolates reported here originated from different poultry farms within and outside the metropolis of Kano State. Post-mortem lesions include haemorrhagic intestinal ulcers, haemorrhagic caecum tonsils, haemorrhagic and inflamed proventriculus, and haemorrhagic trachea, among others (Figure 4). These lesions agree with overt clinical signs reported by poultry handlers such as difficulty in breathing, greenish diarrhoea, weakness, and anorexia with wing and leg paralysis as the most common neurological symptoms among flocks. Haemorrhage at the tip of the proventriculus is highly suggestive of ND [26, 27]. Generally, distributed lesions suggest that the virus is able to infect and replicate in most organs, typical of vNDV as supported by the cleavage site motif of the isolates.

In a previous review report, phylogenetically, the Nigerian genotype XIVa isolates form a cluster with some strains in Niger Republic while genotype XIVb isolates tend to be more closely related to the 2009 isolates from Benin Republic [24]. They further stated that the isolates in genotype XIVa that share the highest nucleotide similarity with those from Niger Republic were all obtained from Sokoto State which shares a direct international border with Niger Republic. Isolates reported here show 99% homology with MT543153 NDV/chicken/Niger/89/2019 (obtained on GenBank as on 15th February, 2022) isolated in 2019 from Niger Republic. It has been reported that importation of poultry in Niger Republic is an informal sector with porous borders between Nigeria and Burkina Faso that allow for the entry of live poultry (chicken and guinea fowl) without prior registration and customs clearance. Informal exportation of poultry from Niger is primarily to Nigeria [28]. This close phylogenetic relationship can be explained by the cross border movements between the two countries which may facilitate the spread of the virus. Live bird trading is commonly practised within West Africa, and birds can be moved over long distances and across porous borders. In Nigeria, environmental factors, high demand, and movement of birds tally with increased incidence of NDV [29, 30]. Sub-genotype XIVb has been isolated in a commercial farm in Nigeria that has vaccinated with unspecified vaccine strain against NDV [21]. With recent isolation of genotype XIVb among flock vaccinated with LaSota and Komarov vaccine strains in Kano State, Nigeria, the spread of this genotype to other regions of the continent may spread vaccine resistance as well.

The F protein is capable of provoking host immune response, and it is necessary for producing neutralizing antibodies against NDV induced by vaccines [19]. Mutations along this gene will impact antibody production which will be heterologous even at the level of F-gene [31]. Percentages of nucleotide identity and aa homology between the study isolates and commercially available vaccines ranges between 81.14 and 84.39% and 0.175–0.211 respectively (Tables 3 and 4). Compared to the commonly used vaccines in Kano State (LaSota and Komarov) with representative KU665482.1 LaSota.71.IR/2016 and KT448901.1 Avian orthoavulavirus 1 strain Komarov (Tables 3 and 4), range is 81.20–81.70%; 0.207–0.210 and 81.65–82.22; 0.201–0.205 respectively indicating considerable diversity [32]. This is similar to reports by [21], based on the analysis of the complete F coding sequences reported, that all the virulent NDV types circulating in Nigeria are shown to be distantly related to currently available vaccine strains in the country.

The evolutionary distance between vaccine and the circulating field strains is an important factor in effective disease control since it explains the continuous occurrence of ND outbreaks despite the extensive poultry vaccination programme which has also been reported in Nigeria [24]. Genomic divergence and antigenic divergence between wild infective strains and vaccine strains are among reported causes of ND vaccine failure [19]. Most commonly used ND vaccine strains including LaSota were developed in the 1950s and 1960s [32] and show considerable degree of genetic divergence from currently circulating vNDV wild strains [31]. LaSota is classified under genotype II, while most commonly reported wild vNDV strains in the 21^st^ century are found among genotype VII [24]. Existing antigenic variations among West Africa strains and the LaSota vaccine may affect its protective efficacy to confer protection against all West African strains [33]; in cases where the LaSota vaccine provided protection against clinical disease, it did not prevent infection and viral shedding [33]. Although all NDV strains belong to one serotype, protection provided by genotype II vaccines against heterologous challenge has been recently under controversy [31] with several reports of LaSota vaccine failing to provide complete protection against morbidity and or mortality during experimental heterologous vNDV challenge necessitating the growing need for development of antigenically matched vaccines to circulating strains [12].

The fusion protein is a major target for the immune response, and immunity raised against this protein is effective in the neutralization of NDV infectivity [34]. In this report, several nt substitutions occur at specific and conserved antigenic sites which in some instances resulted in aa substitution (mutation) (Tables 7–10). The neutralizing epitopes are important in forming antigenic epitopes, and aa substitution in this region induces the formation of neutralizing escape variants [20, 35–37]. The single point K78E mutation of the A2 antigenic epitope seen in all study isolates is due to AAA-AGA nt substitution at codon 232–234 (Table 7) which has been previously reported to induce escape mutation in NDV Beaudette-C clone against MAbs generated using LaSota strains [5]. The role of this mutation in genotype XIVb requires more attention. Though the scope of this research did not involve the generation of K78E mutants to test LaSota efficacy, it is notable from samples and data collected that though the flocks were vaccinated, ND is still reported. The fusion peptide domain between aa residues 117 to 142 is a conserved hydrophobic region located at the amino end of the F1 polypeptide and has been reported to insert into the target membrane to initiate membrane fusion [38, 39]. Phenylalanine (F) residue at position 117 has been reported as a major contributor to neurological symptoms [40, 41]. All study isolates show F117, and paralysis of leg and wings was highly reported among the flocks. Previous report shows that mutation along the fusion peptide domain inhibits fusion affecting syncytia formation, content mixing, and hemifusion [42]. 128-Glycine residue affects folding of the molecule [43]. Among study isolates, I118V, I121V, G1124S, and I135V were observed (Table 5), and the effects of some of these mutations have not been established and require further study.

There is loss of one conserved cysteine residue in study isolate OK491977 Avian orthoavulavirus 1 isolate KN75 due to TGC to TGT nt substitution at codon 1180–1182 causing unique point cysteine (C) to serine substitution at residue (C394S mutation). Cysteine residues have been reported to enhance F1 and F2 disulphide linkages [44].

The fusion gene has three heptad repeat (HR) regions, HRa, HRb, and HRc at positions 143–185, 268–299, and 471–500, respectively [19]. K145, N272Y, S278P, I285K, T288N, N297K, N476T, N479D, E482A, R486N, K494R, and T498S were observed among isolates (Table 6). There is loss of 285-isoleucine residue in isolates OK491973 and OK491975; however, the effect of substitution of hydrophobic isoleucine with hydrophilic lysine at this point on the leucine zipper motif [45] is unknown. Replacing glutamic acid with alanine at position 482 (E482A) has been reported to have little or no effect on the fusogenic activity of the protein [45]. Predicted glycosylation sites on the F gene are well conserved in all isolates, and hence no impairment is expected in viral structure, during viral replication or virulence mediated by differential glycosylation.

Mutations occur over time along the F gene due to response to vaccine or drug pressure [46–48]. In Nigeria, there is report of sub-optimal vaccine dose administration, excess intake of vaccine by flocks, improper handling and storage of vaccines, and indiscriminate and prolonged exposure of poultry to antibiotics for preventive or therapeutic purposes for vaccine preventable infection and disease with evidence of antibiotic residue in poultry products: meat, offals, and eggs [49, 50]. Due to degeneracy of aa, not all nt substitution resulted into aa mutation in this report. However, continuous vaccine and drug pressure may cause mutation at somewhat conserved regions as NDV continuously evolves. Furthermore, these results suggest that there should be improved reporting of ND outbreak among vaccinated flock in Kano State and Nigeria as a whole.

## 5. Conclusion

Virulent NDV genotype XIVb was detected among vaccinated poultry in Kano State, Nigeria. Fusion gene of seven vNDV strains was successfully sequenced, and details are available on NCBI GenBank (OK491971-OK491977). Based on amino acid analysis, several mutations were seen and reported along the F-gene compared to commercially available vaccines. Whether these mutations individually or combined affect antigenicity of the virus remains to be established. Based on nucleotide analysis, all isolates show high degree of antigenic variability from commonly used LaSota and Komarov ND vaccines in Kano State and others commercially available. Although caution is warranted in considering genetic distance between NDV vaccines and the challenge virus as the sole cause of the reported cases of vaccine escape in field report, [51] the isolation and detection of vNDV genotype XIVb among vaccinated flock require further research and especially the role of the A2 antigenic epitope in inducing antibody escape mutation among that sub-genotype. Furthermore, the role of Genotype II NDV strain vaccine pressure on evolutionary change among Class XIVb vNDV require further evaluation. The role of Genotype II NDV strain vaccine pressure on evolutionary change among Class XIVb vNDV require further evaluation. In order to generate genetically matched vaccines for this region, ND surveillance and molecular analysis of circulating strains should be encouraged and reported.

## Figures and Tables

**Table 1:** Primers used for sequence of full F-gene of NDV.

Primer name	Direction	Primer	Location	Product size	References
NDV-F4217F	Forward	5′-TGCGGAGTGTGAAAGTCATCATT-3′	4217-4239	1240 bp	JF966385.1
NDV-F5457R	Reverse	5′-TGCTGAGGCAAACCCTTTGT-3′	5438-5457		

NDV-F5296F	Forward	5′-ATTGGTAGCGGCTTGATCACTG-3′	5296-5317	999 bp	
3′NDV-F6295R	Reverse	5′-CGTTCTACCCGTGTACTGCTCTTT-3′	6272-6295		

**Table 2:** Sample collected, sequence ID, and corresponding accession number.

Sample collection ID	Sequence ID	Isolated specimen voucher	Isolated from	Accession number
PCR/014/200320	Seq 14	F gene KN 14	Broiler	OK491971 Avian Orthoavulavirus 1 Isolate KN 14
PCR/036/250320	Seq 36	F gene KN 36	Broiler	OK491972 Avian Orthoavulavirus 1 Isolate KN 36
PCR/048/300320	Seq 48	F gene KN 48	Broiler	OK491973 Avian Orthoavulavirus 1 Isolate KN 48
PCR/055/310320	Seq 55	F gene KN 55	Broiler	OK491974 Avian Orthoavulavirus 1 Isolate KN 55
PCR/056/310320	Seq 56	F gene KN 56	Broiler	OK491975 Avian Orthoavulavirus 1 Isolate KN 56
PCR/071/020420	Seq 71	F gene KN 71	Layer	OK491976 Avian Orthoavulavirus 1 Isolate KN 71
PCR/075/030420	Seq 75	F gene KN 75	Layer	OK491977 Avian Orthoavulavirus 1 Isolate KN 75

Details of sample ID, collection dates, isolates of study, source, and accession numbers as assigned by NCBI.

**Table 3:** Percentage nucleotide identity between study isolates, traditional vaccine strains, and some isolates reported from West Africa.

NDV isolate	Nucleotide homology (%)						
	OK491971	OK491972	OK491973	OK491974	OK491975	OK491976	OK491977
KU665482.1 LaSota.71.IR/2016	81.58	81.70	81.50	81.44	81.44	81.26	81.20
KT445901.1 Avian orthoavulavirus1strain Komarov	82.10	82.22	82.04	81.86	81.86	81.74	81.65
JF950509.1 Newcastle disease virus strain Mukteswar	83.02	83.14	83.35	83.29	83.17	82.87	82.99
JN872154.1 Avian orthoavulavirus 1 isolate Beaudette C	82.14	82.26	82.18	81.99	81.99	81.81	81.75
JN872151.1 Avian orthoavulavirus 1 isolate Hitchner	81.82	81.94	81.74	81.68	81.68	81.50	81.44
JX316216.1 Newcastle disease virus strain R2B	82.43	82.56	82.50	82.20	82.20	82.08	82.17
EU289028.1 Newcastle disease virus strain VG/GA	81.56	81.68	81.44	81.38	81.38	81.20	81.14
JN872152.1 Avian orthoavulavirus 1 isolate Ulster	84.14	84.39	84.21	84.27	84.03	83.97	83.67
KC987036.1 Newcastle disease virus strain F	81.77	82.02	81.74	81.56	81.68	81.44	81.47
KU058680.1 NDV/Duck/Nigeria/903/KUDU-113/1992	90.78	91.14	91.08	91.14	91.02	91.02	90.78
HF969167.1 NDV/Turkey/Nigeria/N IE 10-082/2011	91.51	91.87	91.69	91.75	91.87	91.57	91.40
HF969143.1 NDV/Chicken/Nigeria/NIE 09-1597/2009	95.79	96.15	95.97	95.91	96.03	95.85	95.55
FJ772449.1 NDV/Avian-913-33Nigeria-2006	88.76	89.00	88.86	88.80	88.67	88.80	88.49
JX390609.1 NDV/Chicken/Togo/A KO18/2009	88.49	88.73	88.55	88.61	88.49	88.55	88.19
HF969175.1 NDV/Chicken/Nigeria/NIE 10-306/2011	88.34	88.58	88.37	88.31	88.31	88.25	88.03
JF966385.1 NDV 2008_Mali_ML007_08	88.76	89.00	88.86	88.80	88.67	88.80	88.49
MT543153.1 NDV/Chicken/Niger/89/2019	98.43	98.68	98.31	98.68	98.68	98.13	98.13

Values calculated from the complete F-gene sequences. Align sequence nucleotide blast showing homology of appropriate % query cover on NCBI blastn suite.

**Table 4:** Estimates of evolutionary divergence (%) between sequences calculated using aa substitutions between study isolates, traditional vaccine strains, and some isolates reported from West Africa.

NDV isolate	Amino acid homology						
	OK491971	OK491972	OK491973	OK491974	OK491975	OK491976	OK491977
KU665482.1 LaSota.71.IR/2016	0.208	0.207	0.207	0.208	0.208	0.210	0.210
KT445901.1 Avian orthoavulavirus1strain Komarov	0.203	0.202	0.201	0.203	0.203	0.205	0.205
JF950509.1 Newcastle disease virus strain Mukteswar	0.193	0.191	0.188	0.188	0.190	0.193	0.091
JN872154.1 Avian orthoavulavirus 1 isolate Beaudette C	0.204	0.202	0.202	0.204	0.204	0.206	0.206
JN872151.1 Avian orthoavulavirus 1 isolate Hitchner	0.205	0.204	0.204	0.205	0.205	0.208	0.207
JX316216.1 Newcastle disease virus strain R2B	0.202	0.200	0.198	0.202	0.202	0.203	0.202
EU289028.1 Newcastle disease virus strain VG/GA	0.209	0.207	0.208	0.209	0.209	0.211	0.211
JN872152.1 Avian orthoavulavirus 1 isolate Ulster	0.178	0.175	0.176	0.175	0.178	0.179	0.182
KC987036.1 Newcastle disease virus strain F	0.207	0.204	0.204	0.207	0.205	0.208	0.207
KU058680.1 NDV/Duck/Nigeria/903/KUD U-113/1992	0.090	0.093	0.094	0.093	0.095	0.095	0.097
HF969167.1 NDV/Turkey/Nigeria/NIE 10082/2011	0.089	0.085	0.087	0.086	0.085	0.088	0.089
HF969143.1 NDV/Chicken/Nigeria/NIE 09-1597/2009	0.043	0.039	0.041	0.042	0.041	0.043	0.045
FJ772449.1 NDV/Avian-91333-Nigeria-2006	0.121	0.118	0.119	0.120	0.121	0.120	0.123
JX390609.1 NDV/Chicken/Togo/AKO18/2 009	0.123	0.120	0.122	0.122	0.123	0.122	0.126
HF969175.1 NDV/Chicken/Nigeria/NIE 10-306/2011	0.126	0.124	0.124	0.125	0.125	0.126	0.128
JF966385.1 NDV 2008_Mali_ML007_08	0.121	0.118	0.119	0.120	0.121	0.120	0.122
MT543153.1 NDV/Chicken/Niger/89/2019	0.016	0.013	0.017	0.013	0.019	0.018	0.095

The number of amino acid substitutions per site between sequences is shown. Analyses were conducted using the Poisson correction model [17]. This analysis involved 24 amino acid sequences. All positions with less than 95% site coverage were eliminated, i.e., fewer than 5% alignment gaps, missing data, and ambiguous bases were allowed at any position (partial deletion option). There were a total of 1662 positions in the final dataset. Evolutionary analyses were conducted in MEGA11 [16].

**Table 5:** Amino acid changes of fusion protein of vNDV study isolates compared with vaccine strains and strains previously reported in Nigeria and West Africa.

ID virus isolate	Amino acid at indicated position on fusion protein																
	Hypervariable region/signal peptide						AGR	Cleavage site					Fusion peptide				
	4	11	15	28	29	31	78	112	113	114	115	116	117	118	121	124	135
^*∗*^KU665482.1 LaSota.71.IR/2016^†^	*R*	*A*	*L*	*P*	*A*	*S*	*K*	*G*	*R*	*Q*	*G*	*R*	*L*	*I*	*I*	*G*	*I*
KT445901.1 Avian orthoavulavirus 1 strain Komarov^†^	—	*T*	—	—	—	—	—	*R*	—	—	*K*	—	*F*	—	—	—	—
JF950509.1 Newcastle disease virus strain Mukteswar^†^	—	*V*	—	*L*	*T*	—	—	*R*	—	—	*R*	—	*F*	—	—	S	—
JN872154.1 Avian orthoavulavirus 1 isolate Beaudette C^†^	—	*V*	—	—	—	—	—	*R*	—	—	*K*	—	*F*	—	—	—	—
JN872151.1 Avian orthoavulavirus 1 isolate Hitchner^†^	—	—	—	—	—	—	—	—	—	—	—	—	—	—	—	—	—
JX316216.1 Newcastle disease virus strain R2B^†^	—	*T*	—	—	—	—	—	*R*	—	—	*K*	—	*F*	—	—	—	—
EU289028.1 Newcastle disease virus strain VG/GA^†^	—	—	—	—	—	—	—	—	—	—	—	—	—	—	—	—	*M*
JN872152.1 Avian orthoavulavirus 1 isolate Ulster^†^	—	*V*	—	—	*T*	—	—	—	*K*	—	—	—	—	—	—	—	—
KC987036.1 Newcastle disease virus strain F^†^	—	—	—	—	—	—	—	—	—	—	—	—	—	—	—	—	—
OK491971 Avian orthoavulavirus 1 isolate KN 14^‡^	*K*	*V*	—	*L*	—	*P*	*R*	*R*	—	*R*	*K*	—	*F*	*V*	*V*	*S*	*V*
OK491972 Avian orthoavulavirus 1 isolate KN 36^‡^	*K*	*V*	—	—	*T*	*P*	*R*	*R*	—	*R*	*K*	—	*F*	*V*	—	—	*V*
OK491973 Avian orthoavulavirus 1 isolate KN 48^‡^	*K*	*V*	—	*L*	*T*	*P*	*R*	*R*	—	*R*	*K*	—	*F*	*V*	—	—	*V*
OK491974 Avian orthoavulavirus 1 isolate KN 55^‡^	*K*	*V*	—	*L*	*T*	*P*	*R*	*R*	—	*R*	*K*	—	*F*	*V*	—	—	*V*
OK491975 Avian orthoavulavirus 1 isolate KN 56^‡^	*K*	*V*	—	*L*	—	*P*	*R*	*R*	—	*R*	*K*	—	*F*	*V*	—	—	*V*
OK491976 Avian orthoavulavirus 1 isolate KN 71^‡^	*E*	*E*	Q	*L*	*T*	*P*	*R*	*R*	—	*R*	*K*	—	*F*	*V*	—	—	*V*
OK491977 Avian orthoavulavirus 1 isolate KN 75^‡^	*K*	*V*	—	*L*	*T*	*P*	*R*	*R*	—	*R*	*K*	—	*F*	*V*	—	—	*V*
KU058680.1 NDV/Duck/Nigeria/903/KUDU-113/1992⁑	*K*	—	—	*L*	*T*	—	—	*R*	—	—	*K*	—	*F*	—	—	—	—
HF969167.1 NDV/Turkey/Nigeria/NIE 10–082/2011⁑	*K*	*V*	—	*L*	*T*	—	*R*	*R*	—	—	*K*	—	*F*	—	—	—	—
HF969143.1 NDV/Chicken/Nigeria/NIE 09–1597/2009⁑	*I*	*V*	—	*L*	*T*	—	*R*	*R*	—	—	*K*	—	*F*	*V*	—	—	*V*
FJ772449.1 NDV/Avian-913-33-Nigeria-2006⁑	*K*	*V*	*P*	*M*	*T*	—	—	*R*	—	—	*K*	—	*F*	—	—	—	—
JX390609.1 NDV/Chicken/Togo/AKO18/2009⁑	*K*	*V*	—	*L*	*T*	—	*R*	*R*	—	*R*	*K*	—	*F*	—	—	—	—
HF969175.1 NDV/Chicken/Nigeria/NIE 10–306/2011^‡^	*K*	*V*	*P*	*M*	*T*	—	—	*R*	—	*R*	*K*	—	*F*	—	—	—	*V*
JF966385.1 NDV 2008 Mali ML007-08	*K*	*V*	*P*	*M*	*T*	—	—	*R*	—	—	*K*	—	*F*	—	—	—	—
MT543153.1 NDV/Chicken/Niger/89/2019	*K*	*V*	—	*L*	*T*	*P*	*R*	*R*	—	*R*	*K*	—	*F*	*V*	—	—	*V*

Variable positions along functional sites in the fusion protein showing point mutation compared with nine commercially available vaccine strains using LaSota KU665482.1 as reference. Mutation patterns vary in some instances among research isolates. ^*∗*^Reference strain. ^†^Vaccine strain. ^‡^Study isolates. ⁑⁑Nigerian isolates. —, no change in the aa compared with the reference; AGR, antigenic region; *R*, arginine; *K*, lysine; *E*, glutamic acid; *I*, isoleucine; *A*, alanine; *T*, threonine; *V*, valine; *L*, leucine; *Q*, glutamine; *P*, proline; *M*, methionine; *G*, glycine; *F*, phenylalanine; *S*, serine.

**Table 6:** Amino acid changes along fusion protein of vNDV study isolates compared with vaccine strains and strains previously reported in Nigeria and West Africa.

ID virus isolate	Amino acid at indicated position on fusion protein												
	HRa	HRb					Conserved cysteine residue	HRc					
	145	272	278	285	288	297	394	476	479	482	486	494	498
^*∗*^KU665482.1 LaSota.71.IR/2016^†^	*K*	*N*	*S*	*I*	*T*	*N*	*C*	*N*	*N*	*E*	*R*	*K*	*T*
KT445901.1 Avian orthoavulavirus 1 strain Komarov^†^	—	—	—	—	—	—	—	—	—	—	*S*	—	—
JF950509.1 Newcastle disease virus strain Mukteswar^†^	*N*	—	—	—	—	—	—	—	*D*	—	*S*	*R*	—
JN872154.1 Avian orthoavulavirus 1 isolate Beaudette C^†^	—	—	—	—	—	—	—	—	—	—	*S*	—	—
JN872151.1 Avian orthoavulavirus 1 isolate Hitchner^†^	—	—	—	—	—	—	—	—	—	—	—	—	—
JX316216.1 Newcastle disease virus strain R2B^†^	—	—	—	—	—	—	—	—	*G*	—	*S*	—	—
EU289028.1 Newcastle disease virus strain VG/GA^†^	—	—	—	—	—	—	—	—	—	—	—	—	—
JN872152.1 Avian orthoavulavirus 1 isolate Ulster^†^	*N*	—	—	—	—	—	—	—	*D*	—	*S*	—	—
KC987036.1 Newcastle disease virus strain F^†^	—	—	—	—	—	—	—	—	—	—	S	—	—
OK491971 Avian orthoavulavirus 1 isolate KN 14^‡^	*N*	*Y*	*P*	—	*N*	—	—	—	*D*	*A*	*N*	*R*	*S*
OK491972 Avian orthoavulavirus 1 isolate KN 36^‡^	N	Y	*P*	—	*N*	—	—	—	*D*	*A*	*N*	*R*	*S*
OK491973 Avian orthoavulavirus 1 isolate KN 48^‡^	*N*	*Y*	*P*	*K*	*N*	*K*	—	—	*D*	*A*	*N*	*R*	*S*
OK491974 Avian orthoavulavirus 1 isolate KN 55^‡^	*N*	*Y*	*P*	—	*N*	—	—	—	*D*	*A*	*N*	*R*	*S*
OK491975 Avian orthoavulavirus 1 isolate KN 56^‡^	*N*	*Y*	*P*	*K*	*N*	—	—	—	*D*	*A*	*N*	*R*	*S*
OK491976 Avian orthoavulavirus 1 isolate KN 71^‡^	*N*	*Y*	*P*	—	*N*	—	—	*T*	*D*	*A*	*N*	*R*	*S*
OK491977 Avian orthoavulavirus 1 isolate KN 75^‡^	*N*	*Y*	*P*	—	*N*	—	S	—	*D*	*A*	*N*	*R*	*S*
KU058680.1 NDV/Duck/Nigeria/903/KUDU-113/1992^‡^	*N*	*Y*	—	—	*N*	—	—	—	*D*	*A*	*S*	*R*	—
HF969167.1 NDV/Turkey/Nigeria/NIE 10–082/2011^‡^	*N*	*Y*	*P*	—	*N*	—	—	—	*D*	*A*	*N*	*R*	*A*
HF969143.1 NDV/Chicken/Nigeria/NIE 09–1597/2009^‡^	*N*	*Y*	*P*	—	*N*	—	—	—	*D*	*A*	*D*	*R*	*A*
FJ772449.1 NDV/Avian-913-33-Nigeria-2006⁑	*N*	*Y*	—	—	*N*	—	—	*S*	*D*	*A*	*S*	*R*	—
JX390609.1 NDV/Chicken/Togo/AKO18/2009	*N*	*Y*	*P*	—	*N*	—	—	—	*D*	*A*	*S*	*R*	—
HF969175.1 NDV/Chicken/Nigeria/NIE 10–306/2011‡	*N*	*Y*	—	—	*N*	—	—	—	*D*	*A*	*S*	*R*	—
JF966385.1 NDV 2008 Mali ML007-08	*N*	*Y*	—	—	*N*	—	—	S	*D*	*A*	*S*	*R*	—
MT543153.1 NDV/Chicken/Niger/89/2019	*N*	*Y*	*P*	—	*N*	—	—	—	*D*	*A*	*N*	*R*	*S*

Variable positions along functional sites in the fusion protein showing point mutation compared with nine commercially available vaccine strains using LaSota KU665482.1 as reference. Mutation patterns vary in some instances among research isolates. ^*∗*^Reference strain. ^†^Vaccine strain. ^‡^Study isolates^.^⁑Previous Nigerian isolates. —, no change in the aa compared with the reference; HR = heptad repeats; *K*: lysine; *N*: asparagine; *Y*: tyrosine; *S*: serine; *P*: proline; *I*: isoleucine; *T*: threonine; *D*: aspartic acid; *G*: glycine; *E*: glutamic acid; *A*: alanine; *R*: arginine.

**Table 7:** Point mutation pattern along fusion protein of study isolates compared to reference LaSota strain KU665482.1.

ID virus isolate	Nucleotide at indicated position along the fusion gene									
	Hypervariable region/Signal peptide							AGR	Cleavage site	
	4		11	15	28	29	31	78	112	113
	Codon	10–12	31–33	43–45	82–87	85–87	TCC	232–234	334–336	337–339
^*∗*^KU665482.1 LaSota.71.IR/2016^†^		AGA	GCA	CTG	CCG	GCA	*C*CC (S31P)	AAA	GGC	AGA
OK491971 Avian orthoavulavirus 1 isolate KN 14^‡^		A*A*A (P4K)	G*T*A (A11V)	CTG	C*T*G (L28P)	GCA	*C*CC (S31P)	A*G*A (K78R)	*A*G*A* (GII2R)	*C*GA (R113)
OK491972 Avian orthoavulavirus 1 isolate KN 36^‡^		A*A*A (P4K)	G*T*A (A11V)	CTG	CCG	*A*CA (A29T)	*C*CC (S31P)	A*G*A (K78R)	*A*G*A* (GII2R)	*C*GA (R113)
OK491973 Avian orthoavulavirus 1 isolate KN 48^‡^		A*A*A (P4K)	G*T*A (A11V)	CTG	C*T*G (L28P)	*A*CA (A29T)	*C*CC (S31P)	A*G*A (K78R)	*A*G*A* (GII2R)	*C*GA (R113)
OK491974 Avian orthoavulavirus 1 isolate KN 55^‡^		A*A*A (P4K)	G*T*A (A11V)	CTG	C*T*G (L28P)	*A*CA (A29T)	*C*CC (S31P)	A*G*A (K78R)	*A*G*A* (GII2R)	*C*GA (R113)
OK491975 avian orthoavulavirus 1 isolate KN 56^‡^		A*A*A (P4K)	G*T*A (A11V)	CTG	C*T*G (L28P)	GCA	*C*CC (S31P)	A*G*A (K78R)	*A*G*A* (GII2R)	*C*GA (R113)
OK491976 Avian orthoavulavirus 1 isolate KN 71^‡^		*GA*A (P4E)	G*A*A (A11E)	C*AG* (L15Q)	C*T*G (L28P)	*A*CA (A29T)	*C*CC (S31P)	A*G*A (K78R)	*A*G*A* (GII2R)	*C*GA (R113)
OK491977 Avian orthoavulavirus 1 isolate KN 75^‡^		A*A*A (P4K)	G*T*A (A11V)	CTG	C*T*G (L28P)	*A*CA (A29T)	*C*CC (S31P)	A*G*A (K78R)	*A*G*A* (GII2R)	*C*GA (R113)

Variable positions along functional sites in the fusion protein showing nucleotide substitution compared with LaSota KU665482.1 vaccine strain as reference. Not all substitution resulted in mutation because of degeneracy of amino acid. Italic positions show substitution site. ^*∗*^Reference strain. ^†^Vaccine strain. ^‡^Study isolates. AGR, antigenic region; A: adenine; G: guanine; C: cytosine; T: thymine.

**Table 8:** Point mutation pattern along fusion protein of study isolates compared to reference LaSota strain KU665482.1.

ID virus isolate	Nucleotide at indicated position along the fusion gene								
	Cleavage site				Fusion peptide				
		114	115	116	117	118	121	124	135
	Codon	340–342	343–345	346–348	349–351	352–354	361–363	370–372	403–405
^*∗*^KU665482.1 LaSota.71.IR/2016^†^		CAG	GGC	CGC	CTT	ATA	ATT	GGT	ATA
OK491971 Avian orthoavulavirus 1 isolate KN 14^‡^		C*G*G (Q114R)	*AAG* (G115K)	CG*T* (R116)	*T*TT (L117F)	*G*T*G* (I118V)	*G*TT (I121V)	*A*GT (G124S)	*G*TA (I135V)
OK491972 Avian orthoavulavirus 1 isolate KN 36^‡^		C*G*G (Q114R)	*AAG* (G115K)	CG*T* (R116)	*T*TT (L117F)	*G*T*G* (I118V)	*G*TT (I121V)	*A*GT (G124S)	*G*TA (I135V)
OK491973 Avian orthoavulavirus 1 isolate KN 48^‡^		C*G*G (Q114R)	*AAA* (G115K)	CG*T* (R116)	*T*TT (L117F)	*G*T*G* (I118V)	*G*TT (I121V)	*A*GT (G124S)	*G*TA (I135V)
OK491974 Avian orthoavulavirus 1 isolate KN 55^‡^		C*G*G (Q114R)	*AAG* (G115K)	CG*T* (R116)	*T*TT (L117F)	*G*T*G* (I118V)	*G*TT (I121V)	*A*GT (G124S)	*G*TA (I135V)
OK491975 Avian orthoavulavirus 1 isolate KN 56^‡^		C*G*G (Q114R)	*AAG* (G115K)	CG*T* (R116)	*T*TT (L117F)	*G*T*G* (I118V)	*G*TT (I121V)	*A*GT (G124S)	*G*TA (I135V)
OK491976 Avian orthoavulavirus 1 isolate KN 71^‡^		C*G*G (Q114R)	*AAA* (G115K)	CG*T* (R116)	*T*TT (L117F)	*G*T*A* (I118V)	*G*TT (I121V)	*A*GT (G124S)	*G*TA (I135V)
OK491977 Avian orthoavulavirus 1 isolate KN 75^‡^		C*G*G (Q114R)	AAG (G115K)	CG*T* (R116)	*T*TT (L117F)	*G*T*G* (I118V)	*G*TT (I121V)	*A*GT (G124S)	*G*TA (I135V)

Variable positions along functional sites in the fusion protein showing nucleotide substitution compared with LaSota KU665482.1 vaccine strain as reference. Not all substitution resulted in mutation because of degeneracy of amino acid. Italic positions show substitution site. ^*∗*^Reference strain. ^†^Vaccine strain. ^‡^Study isolates. A: adenine; G: guanine; C: cytosine; T: thymine.

**Table 9:** Point mutation pattern along fusion protein of study isolates compared to reference LaSota strain KU665482.1.

ID virus isolate	Nucleotide at indicated position along the fusion gene							
	HRa		HRb					Conserved cysteine residue
		145	272	278	285	288	297	394
	Codon	433–435	814–816	832–834	862–864	862–864	889–891	1180–1182
^*∗*^KU665482.1 LaSota.71.IR/2016^†^		AAA	AAC	TCA	ATA	ACT	AAT	TGC
OK491971 Avian orthoavulavirus 1 isolate KN 14^‡^		AA*C* (K145N)	*T*AC (N272Y)	*C*CA (S278P)	ATA	A*AA* (T288N)	AAT	TGC
OK491972 Avian orthoavulavirus 1 isolate KN 36^‡^		AA*C* (K145N)	*T*AC (N272Y)	*C*CA (S278P)	ATA	A*AA* (T288N)	AAT	TGC
OK491973 Avian orthoavulavirus 1 isolate KN 48^‡^		AA*C* (K145N)	*T*AC (N272Y)	*C*CA (S278P)	AAA (I285K)	A*AA* (T288N)	AA*A* (N297K)	TGC
OK491974 Avian orthoavulavirus 1 isolate KN 55^‡^		AA*C* (K145N)	*T*AC (N272Y)	*C*CA (S278P)	ATA	A*AA* (T288N)	AAT	TGC
OK491975 Avian orthoavulavirus 1 isolate KN 56^‡^		AA*C* (K145N)	*T*AC (N272Y)	*C*CA (S278P)	AAA (I285K)	A*AA* (T288N)	AAT	TGC
OK491976 Avian orthoavulavirus 1 isolate KN 71^‡^		AA*C* (K145N)	*T*AC (N272Y)	*C*CA (S278P)	ATA	A*AA* (T288N)	AAT	TGC
OK491977 Avian orthoavulavirus 1 isolate KN 75^‡^		AA*C* (K145N)	*T*AC (N272Y)	*C*CA (S278P)	ATA	A*AA* (T288N)	AAT	TG*T* (C394S)

Variable positions along functional sites in the fusion protein showing nucleotide substitution compared with LaSota KU665482.1 vaccine strain as reference. Not all substitution resulted in mutation because of degeneracy nature of amino acid. Italic positions show substitution site. ^*∗*^Reference strain. ^†^Vaccine strain. ^‡^Study isolates. HR: heptad repeats; *A*: adenine; *G*: guanine; *C*: cytosine; *T*: thymine.

**Table 10:** Point mutation pattern along fusion protein of study isolates compared to reference LaSota strain KU665482.1.

ID virus isolate	Nucleotide at indicated position along the fusion gene						
	HRc						
		476	479	482	486	494	498
	Codon	1426–1428	1435–1437	1444–1446	1456–1458	1480–1482	1492–1492
KU665482.1 LaSota.71.IR/2016^†^		AAT	AAT	GAC	AGA	AAA	ACA
OK491971 Avian orthoavulavirus 1 isolate KN 14^‡^		AAT	*G*A*C* (N479D)	G*CA* (E482A)	A*AC* (R486N)	A*G*A (K494R)	*T*CA (T498S)
OK491972 Avian orthoavulavirus 1 isolate KN 36^‡^		AAT	*G*A*C* (N479D)	G*CA* (E482A)	A*AC* (R486N)	A*G*A (K494R)	*T*CA (T498S)
OK491973 Avian orthoavulavirus 1 isolate KN 48^‡^		AAT	*G*A*C* (N479D)	G*CA* (E482A)	A*AC* (R486N)	A*G*A (K494R)	*T*CA (T498S)
OK491974 Avian orthoavulavirus 1 isolate KN 55^‡^		AAT	*G*A*C* (N479D)	G*CA* (E482A)	A*AC* (R486N)	A*G*A (K494R)	*T*CA (T498S)
OK491975 Avian orthoavulavirus 1 isolate KN 56^‡^		AAT	*G*A*C* (N479D)	G*CA* (E482A)	A*AC* (R486N)	A*G*A (K494R)	*T*CA (T498S)
OK491976 Avian orthoavulavirus 1 isolate KN 71^‡^		A*C*T (N476T)	*G*A*C* (N479D)	G*CA* (E482A)	A*AC* (R486N)	A*G*A (K494R)	*T*CA (T498S)
OK491977 Avian orthoavulavirus 1 isolate KN 75^‡^		AAT	*G*A*C* (N479D)	G*CA* (E482A)	A*AC* (R486N)	A*G*A (K494R)	*T*CA (T498S)

Variable positions along functional sites in the fusion protein showing nucleotide substitution compared with LaSota KU665482.1 vaccine strain as reference. Not all substitution resulted in mutation because of degeneracy nature of amino acid. Italic positions show substitution site. *∗*Reference strain. ^†^Vaccine strain. ^‡^Study isolates. HR: heptad repeats; A: adenine; G: guanine; C: cytosine; T: thymine.

## Data Availability

The F-genes of all seven vNDV detected and characterized during this research are available with no restriction on NCBI database with their corresponding accession numbers.

## Supplementary Materials

Supplementary Materials Sixty-one representative NDV F-gene sequences of different genotypes and sub-genotypes isolated from Nigeria and Africa downloaded from GenBank were used for phylogenetic tree and molecular analysis of the study isolates. The file is compatible with Bioedit or MEGA.
